# Characterisation of Iron Content and Speciation in Australian Eggs

**DOI:** 10.3390/foods15142452

**Published:** 2026-07-10

**Authors:** Meg Willans, Gaewyn Ellison, Evelyn S. Innes, Jeremy L. Wykes, Pria Ramkissoon, Simon A. James, Mark J. Hackett, Natalie K. Morgan

**Affiliations:** 1 School of Molecular and Life Sciences, Curtin University, Perth, WA 6102, Australia; meg.willans@postgrad.curtin.edu.au (M.W.); gaewyn.ellison@curtin.edu.au (G.E.); evelyn.innes@postgrad.curtin.edu.au (E.S.I.); 2 Curtin Medical Research Institute, Curtin University, Perth, WA 6102, Australia; 3 Australian Synchrotron, ANSTO Melbourne, 800 Blackburn Road, Clayton, VIC 3168, Australia; jeremyw@ansto.gov.au (J.L.W.); ramkissp@ansto.gov.au (P.R.); sjames@ansto.gov.au (S.A.J.)

**Keywords:** eggs, iron, haem, egg yolk, egg albumen

## Abstract

The quantity and bioavailability of iron (Fe) in commercial chicken eggs have been subject to ongoing debate. Understanding the chemical form of Fe in eggs, and how different laying conditions or cooking environments may alter chemical form is important to guide future studies of Fe bioavailability. To address these unanswered questions, this study aimed to accurately quantify and characterise Fe speciation (including haem and non-haem Fe) in egg yolk, albumen and whole eggs (mixed yolk and albumen), in both raw and cooked eggs. Eggs were obtained from four different hen housing systems: free-range, cage, barn and organic. Total Fe was measured using microwave plasma atomic emission spectrometry, and X-ray absorption near-edge structure spectroscopy was used to quantify the relative proportions of different chemical forms of Fe (Fe speciation). These analyses were conducted on raw albumen, yolk, and whole egg samples (combined yolk and albumen) from eggs produced across all housing systems, as well as on baked and boiled albumen, yolk, and whole egg samples from free-range eggs. Haem Fe was not detected by the analytical methods used, confirming that eggs are not a nutritionally relevant source of haem Fe. Mixing yolk and albumen alters Fe speciation, decreasing relative phosphate coordination of Fe and increasing Fe associated with protein carboxylate and chloride groups. Subsequent baking causes a significant reduction in carboxylate- and chloride-bound Fe, accompanied by an increase in sulfur-bound Fe. Boiling eggs was found to have minimal effects on Fe speciation. Despite contributing little Fe, albumen plays an important role in modulating Fe speciation, which may subsequently impact bioavailability. Cooking eggs changes the Fe speciation, particularly increasing the amount of Fe–S coordination, which should be taken into consideration for future study design when assessing Fe bioavailability.

## 1. Introduction

Iron (Fe) plays an integral role in numerous metabolic processes, such as oxygen transport, deoxyribonucleic acid synthesis, and electron transport [1,2]. In addition to its role in haemoglobin synthesis, Fe is a vital component of approximately 200 enzymes that support normal cellular function and act as a catalyst in numerous biochemical reactions [3]. Fe deficiency is the most prevalent micronutrient deficiency worldwide and accounts for nearly half of all anaemia cases, affecting approximately one quarter of the global population [4]. In Australia, over 22% of women have depleted Fe stores [5]. Fe deficiency is associated with impaired cognitive performance, reduced physical capacity, compromised immune function, and adverse pregnancy outcomes [6,7]. These public health implications underscore the need for a more comprehensive understanding of Fe sources and bioavailability [8,9].

Dietary Fe occurs in three forms: ferrous Fe (Fe^2+^), ferric Fe (Fe^3+^), and haem Fe (Fe^2+^ chelated to haem). Haem Fe (organic form of Fe) is preferentially absorbed in the human gastrointestinal tract [10]. Animal-based food sources are rich in haem Fe; the Fe from these sources is highly bioavailable as it is bound into haemoglobin, myoglobin, cytochromes and Fe-containing haem-enzymes [11,12]. Non-haem Fe (inorganic form of Fe), present in plant-based foods, fortified products, and animal-derived foods, exhibits lower and more variable absorption. At physiological pH, non-haem Fe exists primarily as Fe^3+^ and must be reduced to Fe^2+^ for absorption, adding an additional regulatory step [13]. The bioavailability of non-haem Fe is determined by the balance between absorption enhancers (i.e., ascorbic acid) and inhibitors (i.e., phytates, polyphenols, and certain proteins) and the Fe status of the individual [14,15]. Although eggs do not contain a high concentration of haem Fe, there is evidence they may contain a haem fraction, as multiple studies have reported activity of the haem-containing enzyme catalase [16,17,18].

There remains a lack of consensus regarding both the quantity and bioavailability of Fe in eggs. Reported Fe concentrations in eggs vary considerably across the literature; for example, yolk and albumen values of 2.73 and 0.08 mg/100 g [19], 4.9 and 0.1 mg/100 g [20], 7.10 and 2.10 mg/100 g [21], respectively, have been presented. There are also conflicting views regarding whether Fe intake is optimised through consumption of the yolk, albumen, or the whole egg. For example, Azmandian et al. [22] and Kobayashi et al. [23] observed that consumption of egg albumen increased serum Fe and haemoglobin concentration in anaemic patients, but Hallberg et al. [24] concluded that eating whole eggs reduced Fe absorption by 27%, attributed to the inhibiting effects of albumen. Fe bioavailability differs substantially between egg yolk and albumen due to differences in Fe concentration, speciation, and protein interactions. Egg Fe is predominantly bound to the phosphoprotein phosvitin within the yolk, with binding occurring via Fe^3+^ coordination to phosphate functional groups of the protein [25,26]. The resistance of phosvitin to digestion by human proteolytic enzymes [26] restricts Fe solubility, thus limiting yolk Fe bioavailability [27]. Egg albumen contains little Fe but influences Fe speciation due to its high protein content, particularly ovotransferrin [28], which binds to Fe ions (predominantly through coordination to amino acid residues aspartate, histidine, tyrosine). A lack of understanding about the factors influencing the strength of Fe–protein interactions has limited the establishment of effective strategies to increase non-haem Fe bioavailability [29].

The structural and chemical changes induced by cooking affect Fe bioavailability in eggs. Cooking denatures proteins and some antinutritional factors and promotes lipid oxidation [30,31,32]. Partial denaturation during cooking could release Fe bound to protein complexes, but high temperatures and prolonged cooking can induce the formation of insoluble Fe-protein or Fe-lipid complexes, reducing Fe bioavailability [33,34]. This was illustrated by Réhault-Godbert et al. [31] who observed that calcium, potassium, and selenium concentrations declined exponentially with increasing boiling intensity, from raw to soft- and hard-boiled eggs. Cobbinah-Sam and Ekaette [34] recommend that nutrient retention is optimised in eggs when cooking temperatures are maintained between 62 and 85 °C. Moreover, Frances et al. [35] observed that Fe concentration was higher in fried eggs (3.30 mg/100 g) compared to boiled (2.90 mg/100 g) and raw eggs (3.10 mg/100 g). This can be attributed to greater moisture loss and limited mineral leaching during frying relative to boiling, which promotes Fe loss into water. Combining yolk and albumen during mechanical disruption or heating could allow Fe from the yolk to be sequestered by albumen proteins, potentially altering Fe solubility, analytical extractability, and intestinal absorption. However, there remains a scarcity of research examining the effects of egg mixing followed by cooking on Fe content and bioavailability.

In Australia, commercial eggs are produced by laying hens housed in free-range, barn or cage housing systems, with free-range eggs accounting for nearly 60% of egg sales [36]. An additional market segment is organic eggs, produced by free-range hens fed certified organic grain and reared on pesticide-, herbicide- and fertiliser-free pasture, without routine antibiotic use and at lower stocking densities [37]. Hen housing systems can influence egg mineral content due to differences in diet composition, environmental and pathogen exposure, hen physical activity, feed intake consistency, and stress, all of which affect mineral absorption and metabolism [38]. There is a lack of clarity regarding which housing system produces eggs with the highest Fe content and bioavailability. For example, Heflin et al. [39] reported no effect of housing system (cage, barn, or free-range) on whole-egg Fe content, and Küçükyılmaz et al. [38] similarly observed no difference in whole-egg Fe content between organic and conventional housing, whereas Sekeroglu et al. [40] reported higher total Fe concentrations in eggs from barn-reared hens compared with cage and free-range systems.

Accurate characterisation of Fe speciation in foods presents significant analytical challenges due to the wide range of chemical forms of Fe present in biological samples and the absence of a single analytical method capable of detecting all chemical forms of Fe [41,42]. While haem and non-haem Fe can be quantified using various biochemical assays [43,44], these methods may alter non-haem Fe oxidation state and coordination chemistry. Often Fe is labile, allowing for Fe speciation to be easily perturbed during sample preparation [45]. X-ray absorption near-edge structure (XANES) spectroscopy is a technique that is increasingly being used in the biological sciences to characterise metal ion speciation and is capable of detecting a wide range of Fe species that exist in biological samples [46,47,48]. Further, as XANES spectroscopy is a direct analytical method that does not require chemical reagents, reactions or complex sample preparation, there are fewer issues with sample-preparation-induced alterations to Fe speciation (such as those that can occur when undertaking chemical Fe assay as described above). XANES spectroscopy measures the energy required to excite a core electron to an unoccupied molecular orbital, and the resulting spectra are inherently sensitive to the metal ion coordination environment, providing a fingerprint of metal speciation [46,47,48]. Through the development of a spectral library of standard solutions and model compounds, the speciation of a metal ion in an unknown sample can be assessed (often through approaches such as linear combination fitting, LCF). To the best of our knowledge, XANES spectroscopy has not previously been used to study Fe speciation in chicken eggs; however, it has previously been used to study Fe speciation in egg proteins such as phosvitin [49,50], as well as other biological tissues such as plant tissue [51], brain tissue [52] and worms (*C. elegans*) [53].

The aim of this study was to characterise haem and non-haem Fe in egg yolk, albumen and whole eggs (combined yolk and albumen), in both raw and cooked (baked and boiled) eggs, and to assess if housing conditions (free-range, barn, caged, organic) influence total Fe or Fe speciation. This information may be applied to the experimental design of future studies aiming to inform dietary guidance for individuals affected by Fe deficiency and develop evidence-based cooking strategies to improve Fe bioavailability in eggs.

## 2. Materials and Methods

### 2.1. Egg Preparation

Eggs were sourced from a local egg producer (Golden Eggs, Western Australia) on the day of lay from three different hen housing systems: free-range (*n* = 48), cage (*n* = 36) and barn (*n* = 36). All eggs for the free-range, barn and caged conditions were obtained from 40-week-old Hy-Line hens, to eliminate potential confounding effects of hen age or strain on egg Fe concentration. Organic eggs (*n* = 36) were purchased from a local supermarket, as no egg producers supply eggs from all four housing systems. It is expected that data variation is likely to exist between the organic eggs and free-range, barn and caged eggs due to the organic eggs not being supplier-, age- and breed-matched.

*Preparation of Raw Whole Eggs*—Six eggs from each housing system were extracted from the shell and individually blended using a small domestic electric whisk to combine the yolk and albumen. The blended eggs were placed in 50 mL Falcons and frozen at −80 °C, then freeze-dried to constant weight.

*Preparation of Raw Yolks and Albumen*—Using six eggs from each housing system, yolks were separated from the albumen and mixed using a small domestic hand whisk. The yolks and albumen were placed in separate 50 mL Falcons, frozen at −80 °C, and then freeze-dried to constant weight.

*Preparation of Baked Eggs*—Six blended whole eggs plus yolks and albumen from a further six eggs per housing system were prepared as above, placed in silicone muffin pans lined with baking paper, and baked at 120 °C for 15 min. Once baked, eggs, yolks and albumen were freeze-dried to constant weight.

*Boiled Eggs*—Six free-range eggs were placed in a saucepan with enough water to cover them and boiled for ten minutes on a gas stove. After boiling, cooling and peeling, yolks were separated and freeze-dried to constant weight.

### 2.2. Microwave Plasma Atomic Emission Spectrometry

Samples (~1 g) of raw freeze-dried whole eggs (*n* = 24 total with *n* = 6 per housing system), raw freeze-dried yolks (*n* = 24 total, with *n* = 6 per housing systems), and raw freeze-dried albumen (*n* = 24 total, with *n* = 6 for each housing system), were digested with HNO_3_ (30 mL, 70%) at 120–150 °C for 1.0–1.5 h, until brown fumes were replaced with white fumes. Samples were subsequently digested with H_2_O_2_ (5 mL, 30%) at 80–90 °C for 0.5–1.0 h; once bubbling had subsided and the solution was almost colourless and transparent, samples were made to volume (25 mL) with dilute HNO_3_ (2–3%). Sample solutions were transferred to 15 mL Falcon tubes for microwave plasma atomic emission spectroscopy (MP-AES) analysis. MP-AES analysis was conducted with an Agilent 4210 MP-AES (Mulgrave, Victoria, Australia) instrument, with Fe emission measured at 371.993 nm, with an uptake time of 120 s to completely flush the previous sample and 15 s of stabilisation. Between sets of samples, blanks that were digested simultaneously with samples were run to flush the system, to prevent contamination and to identify if contamination from the digestion process was present. Measurement of 7 replicate blanks was used to assess the minimum limit of detection (3.3σ × standard deviation) and minimum limit of quantification (10σ × standard deviation), which were determined as 0.02 ppm and 0.06 ppm, respectively. Quantification of Fe in the egg samples was performed using a calibration curve constructed from 1 to 100 ppm. Fe calibration standards were prepared via serial dilution of a commercial Fe standard (10,042 +/− 30 µg/mL Fe in 5% HNO_3_, Inorganic Ventures, Virginia). A certified reference material was not used.

### 2.3. X-Ray Absorption Spectroscopy

Fe K-edge XANES spectra of egg samples and reference compounds (model Fe metallo-proteins and standard solutions) were collected at the Australian Synchrotron medium-energy X-ray 1 absorption spectroscopy (MEX1) beamline. The MEX1 beamline is equipped with a bend magnet and Si(111) double crystal monochromator. Standard solutions were measured as frozen solutions (~10 mM Fe concentration, ratio of ~10:1 ligand to Fe) at 12–15K with a liquid Helium cryostat. The list of standard solutions and model Fe metallo-proteins and compounds is as follows: Fe(SO_4_)_aq_, Fe_2_(SO_4_)_3aq_, FeCl_3aq_, Fe_2_(SO4)_3aq_ + excess NaCl, Fe_2_(SO_4_)_3aq_ + excess Na-citrate, Ferritin, Cytochrome C, Haemoglobin, Ferredoxin, Fe_2_O_3_, Fe(III) phytate. All chemical reagents were purchased from Sigma-Aldrich (St. Louis, MO, USA). With the exception of standard solutions, all other samples were analysed as pressed powder pellets at room temperature in a back-filled He environment. Powders of model Fe metallo-proteins and Fe oxides were diluted with cellulose, finely ground and pressed into 7 mm diameter pellets, while pellets for egg samples were prepared neat.

For all samples, fluorescence emission spectra were recorded with a Vortex-ME4 4-element silicon drift detector oriented perpendicular to the incident beam. An Fe foil was placed downstream of the sample was measured simultaneously for energy calibration, with foil spectra recorded in transmission with gas ionisation chambers (energy calibration set to 7112 eV for the first inflexion point of Fe foil). Spectra were recorded with an X-ray beam size of approximately 2 mm × 1 mm. XANES spectra were collected from 6912 to 8000 eV, with energy increments of 0.006–0.03 eV, with slew scanning across the white line.

Although studying Fe speciation was the focus of this study, the full X-ray emission spectrum was recorded at an incident energy of 13,500 eV to help identify the range of elements present in egg yolk and egg albumen. As Zn was also shown to be relatively abundant in eggs, Zn XANES spectra of egg samples were also recorded (in triplicate), across the energy range 9456–9891 eV, with energy increments of 0.03–0.06 eV, with slew scanning across the white line. Reference spectra from prior publications [54,55] were used for fitting the Zn XANES spectra. As the focus of this study was Fe, Zn data will not be discussed in the main manuscript, but are presented in Supporting Information Figure S2.

### 2.4. Synthesis of Fe(III) Phytate

Fe(III) phytate was synthesised based on the procedure described by [56]. Phytic acid sodium salt hydrate (0.202 g, 0.3 mmol, 1 eq.) was dissolved in deionised water (15 mL) and adjusted to pH 7–8 with dilute HCl (1 M). The solution was heated at 50 °C for two hours with stirring. Fe(III) chloride hexahydrate (0.496 g, 1.8 mmol, 6 eq.) was dissolved in a 1:1 solution of deionised water:ethanol (15 mL) and added dropwise to the phytic acid solution, forming a light brown precipitate. The reaction was stirred for a further hour at 50 °C, then quenched in an ice bath. The precipitate was filtered and washed with a hot (50 °C) 1:1 deionised water:ethanol solution (3 × 15 mL), then allowed to air dry.

### 2.5. Data Analysis

Spectra were processed in Larch [57]. Spectra were pre-edge subtracted and normalised to an edge jump of 1. Linear combination fitting (LCF) was used to fit sample spectra with reference spectra to estimate the relative proportions of Fe species. Spectra were fit in the range 7085–7225 eV. We have expanded the LCF methods section to read as: The initial choice of reference spectra to include in fitting was based on literature knowledge of Fe coordination environments likely to be found in eggs. Reference spectra were excluded from the fit if they were not found to contribute above 1% of the fit and residuals were inspected to confirm an absence of features/structure post-fitting. To avoid over-fitting, fits were limited to a linear combination of 5 reference spectra. Uncertainty estimates were evaluated for goodness of fit.

Statistical analysis was performed using the SciPy Statistics Python library (v 1.10.0) [58]. For analysis with three or more experimental groups, a One-Way ANOVA was performed, and if the ANOVA indicated a significant main effect, post hoc multiple comparison testing was then undertaken with Tukey’s test. For experiments with only two comparisons, a Student’s *t*-test was used. Due to the relatively small number of biological replicates, data normality and homogeneity were not assessed. Statistical significance was indicated at the 95% confidence interval. All error bars are presented as the standard error of the mean.

## 3. Results

### 3.1. Determination of Total Egg Fe Content and Comparison Between Housing Conditions (Free-Range, Barn, Caged, Organic)

**Figure 1:**
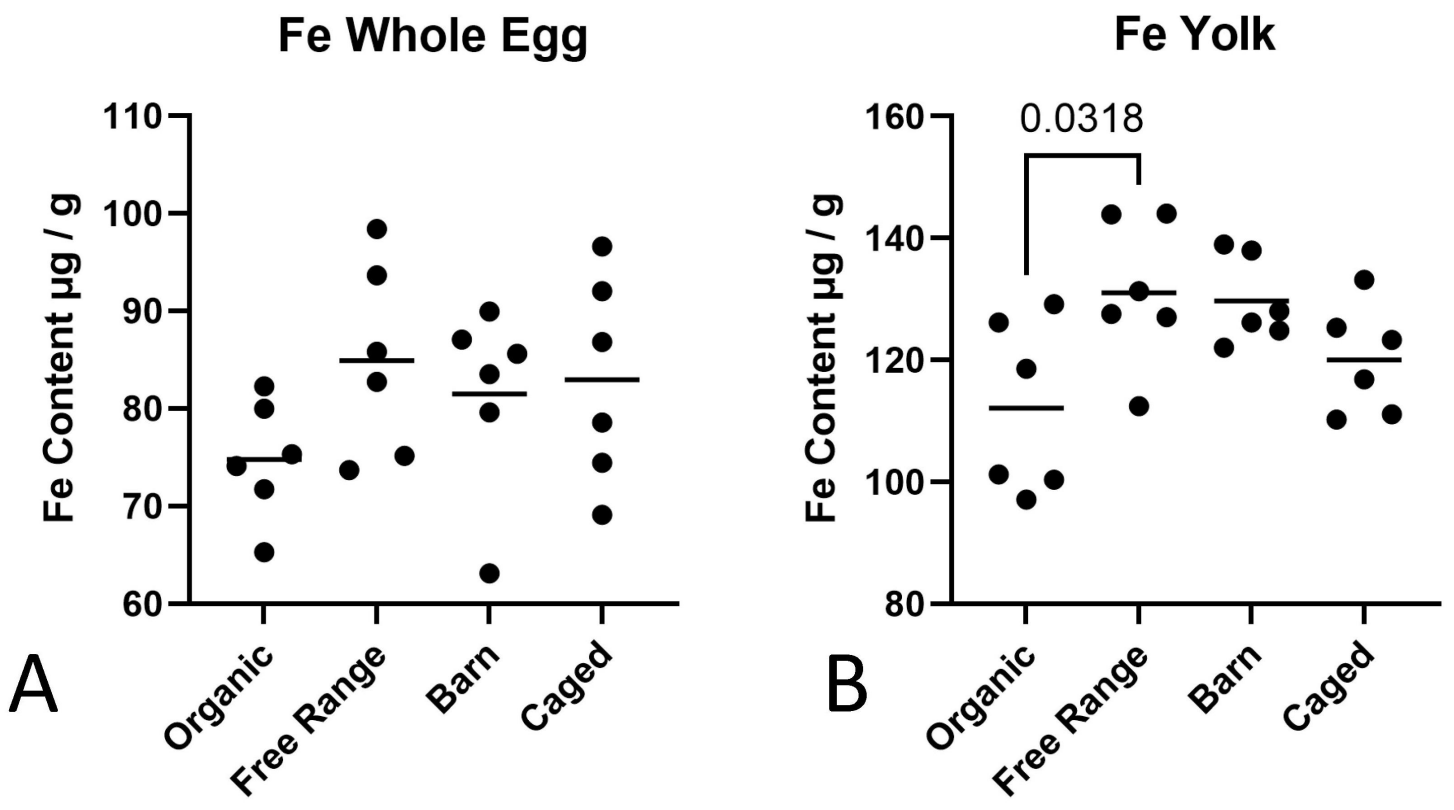
Total Fe content of freeze-dried (**A**) whole eggs and (**B**) egg yolks, from different housing conditions (organic, free-range, barn, caged). There was no significant effect of housing condition on total Fe content of whole eggs; however, a significant effect was observed for the total Fe content of yolks (*p* = 0.022, One Way ANOVA). Multiple comparison testing revealed a significant difference in total Fe content of egg yolks between organic and free-range eggs.

The total Fe composition of freeze-dried whole egg, egg yolks and egg albumen was determined by MP-AES, as highlighted above. The Fe content of egg albumen was below the minimum detection limits of the MP-AES instrumentation. As shown in Figure 1, the average Fe content of whole eggs was 85, 83, 81, and 75 µg of Fe per g of freeze-dried whole egg for free-range, barn, caged and organic housing systems, respectively. In egg yolk, the amount of Fe was found to be 131, 130, 121 and 112 µg of Fe per g of freeze-dried egg yolk for free-range, barn, caged and organic housing systems, respectively. Statistical analysis (One-Way ANOVA) revealed no significant difference in whole egg Fe content between the housing systems. However, a significant effect of housing system was observed for total Fe in egg yolk (*p* = 0.022); multiple comparison testing revealed that there was significantly more Fe in free-range egg yolk (131 µg/g) compared to organic egg yolk (112 µg/g).

### 3.2. Development of an Fe XANES Spectral Library to Characterise Fe Speciation in Egg Samples

**Figure 2:**
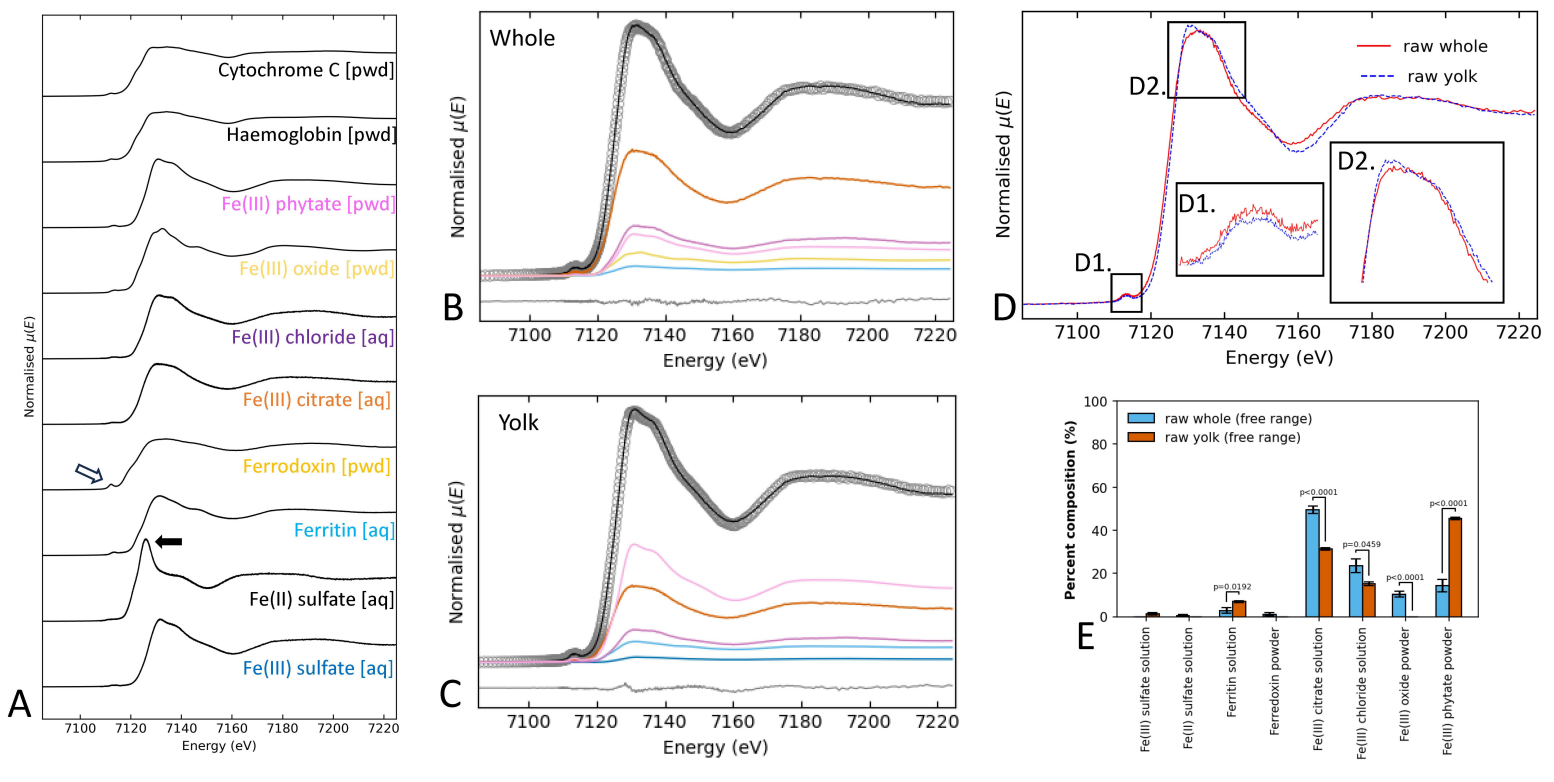
Development of a XANES spectral library to model Fe speciation in eggs. (**A**) XANES spectra of standard solutions and model Fe metallo-proteins. (**B**) Representative example of LCF of K-edge XANES spectra of freeze-dried raw whole egg sample, and (**C**) freeze-dried raw egg yolk. (**D**) Overlay of examples of Fe K-edge XANES spectra of freeze-dried whole egg and yolk spectra. Insets (**D1**,**D2**) show a close-up of the (**D1**) pre-edge and (**D2**) white line feature, showing identical pre-edges in both spectra, but decreased peak intensity and broader white line feature (weakened high-energy shoulder feature) in the whole egg spectrum compared to the egg yolk spectrum. (**E**) Statistical analysis of differences in Fe speciation between raw whole egg and egg yolk, determined from LCF. Error bars are reported as the standard error of the mean.

To characterise Fe speciation in eggs, an Fe XANES spectral library was developed from standard solutions of Fe complexes, Fe oxides and Fe metallo-proteins. Figure 2A presents the Fe K-edge XANES spectra of the spectral library. A representative example showing application of the library to linear combination fitting (LCF) of whole egg and egg yolk is shown in Figure 2B and Figure 2C, respectively. In general, the XANES spectra of the model library resemble spectra already published in the literature for similar compounds. Notable features include the 3.4 eV shift in white line energy between Fe(II) complexes (7123.5 eV) and Fe(III) complexes (7126.9 eV), as shown by the black arrow in Figure 2B. Other features of note include: the increased intensity and lower energy feature of ferredoxin (white arrow), broader (less sharp) appearance of the white line in spectra of haem-proteins, the lower white line intensity of Fe oxides, and the high energy shoulder on the white line in Fe(III) phytate and Fe(III) chloride standard solution. Not unexpectedly, Fe(III) coordinated to phosphate groups in Fe(III) phytate presents a very similar spectrum to those published in the literature for Fe(III) phosvitin [49,50].

**Figure 3:**
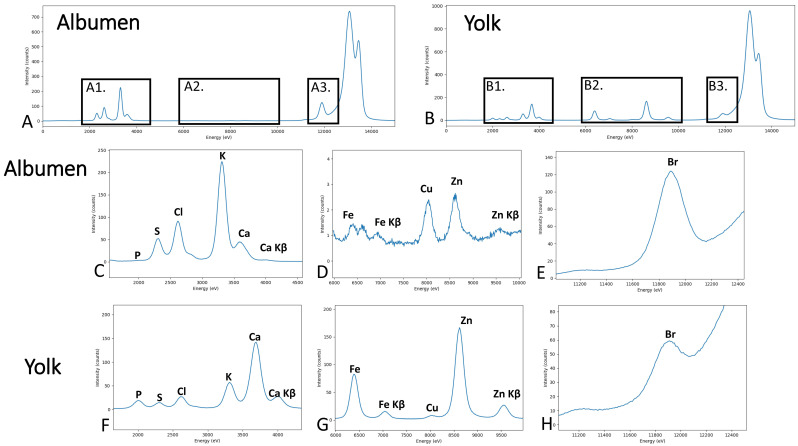
Representative examples of Fe K-edge X-ray emission spectra from freeze-dried (**A**) egg albumen and (**B**) egg yolk. Close-up views of key emission lines for insets (**A1**–**A3**) are shown in panels (**C**–**E**), and insets for (**B1**–**B3**) are shown in panels (**F**–**H**). All emission lines are Kα unless otherwise denoted as Kβ. Egg albumen displayed substantially greater content of S, Cl, K and Br, while egg yolks displayed substantially more P, Ca, Fe, Cu, and Zn. Fe, Cu, and Zn were not above the background signal levels in egg albumen.

Linear combination fitting (Figure 2B,C) indicate that Fe(III) complexes were present in the egg samples [modelled by Fe(III) sulfate, Fe(III) citrate, and Fe(III chloride)], along with Fe sulfur complexes [modelled by ferredoxin], Fe phosphate complexes [modelled by Fe(III) phytate], Fe oxides/hydroxides [modelled by ferritin and Fe(III)oxide]. Importantly, Fe in haem proteins, modelled by haemoglobin and cytochrome C, were not detected in any of the samples analysed. The observation of Fe(III) chloride complexes in the egg samples was somewhat unexpected, but the collection of the full X-ray fluorescence emission spectrum from egg yolks and egg albumen revealed abundant chloride, particularly in egg albumen (Figure 3).

### 3.3. Comparison of Fe Speciation Between Egg Yolk and Whole Egg

Following the development of the Fe K-edge XANES spectral library, linear combination fitting (LCF) was used to characterise Fe speciation in raw egg yolks and whole eggs (combined yolk and white). As can be seen in Figure 2C,E, the major form of Fe in egg yolk was identified to resemble Fe(III) phosphate complexes, which were modelled by Fe(III) phytate. Fe coordination resembling Fe(III) coordinated with phosphates was found to contribute to 45% of the total Fe in egg yolk (eggs from a free-range egg housing system). Other chemical forms of Fe observed in the raw egg yolk included Fe(III) complexed with carboxylate groups [modelled by Fe(III) citrate] (31% on average), Fe(III) complexed to chloride (15% on average) and Fe in a coordination environment resembling Fe bound to ferritin (7% on average). A representative example of LCF of an Fe XANES spectrum of egg yolk is presented in Figure 2C, highlighting the dominant contribution from Fe phosphate complexes.

Fe was not detected in egg albumen samples using XANES spectroscopy; however, as it was detected in the whole eggs, it is therefore assumed that the whole egg Fe signal comes from Fe within the egg yolk. Interestingly, a significant difference in Fe speciation was observed in the whole egg spectra relative to egg yolk spectra (i.e., the mixing of egg yolks and egg albumen alters the Fe coordination environment that was originally present in the egg yolk). The speciation of Fe in whole eggs is presented in Figure 2B,E, with a direct overlay of Fe XANES spectra from egg yolk and whole egg shown in Figure 2D.

The specific differences in Fe speciation between egg yolks and whole eggs are summarised as follows: in raw whole egg, there is a significant increase in Fe coordination with carboxylate groups [31% to 50%, modelled by Fe(III) citrate in this study, but contributions from a variety of organic acids and protein side chains is possible]. The increased speciation of Fe resembling coordination with carboxylate groups occurred concomitantly with a decrease in speciation of Fe resembling coordination to phosphates (45% to 14%). Fe chloride complexes were significantly more abundant in whole egg compared to egg yolks (15% to 24%). Interestingly, Fe in a form resembling Fe coordinated to ferritin was not detected in the whole egg samples; however, Fe oxides were present at approximately the same amount as ferritin in egg yolk (10%).

### 3.4. Comparison of Fe Speciation Between Different Cooking Methods

**Figure 4:**
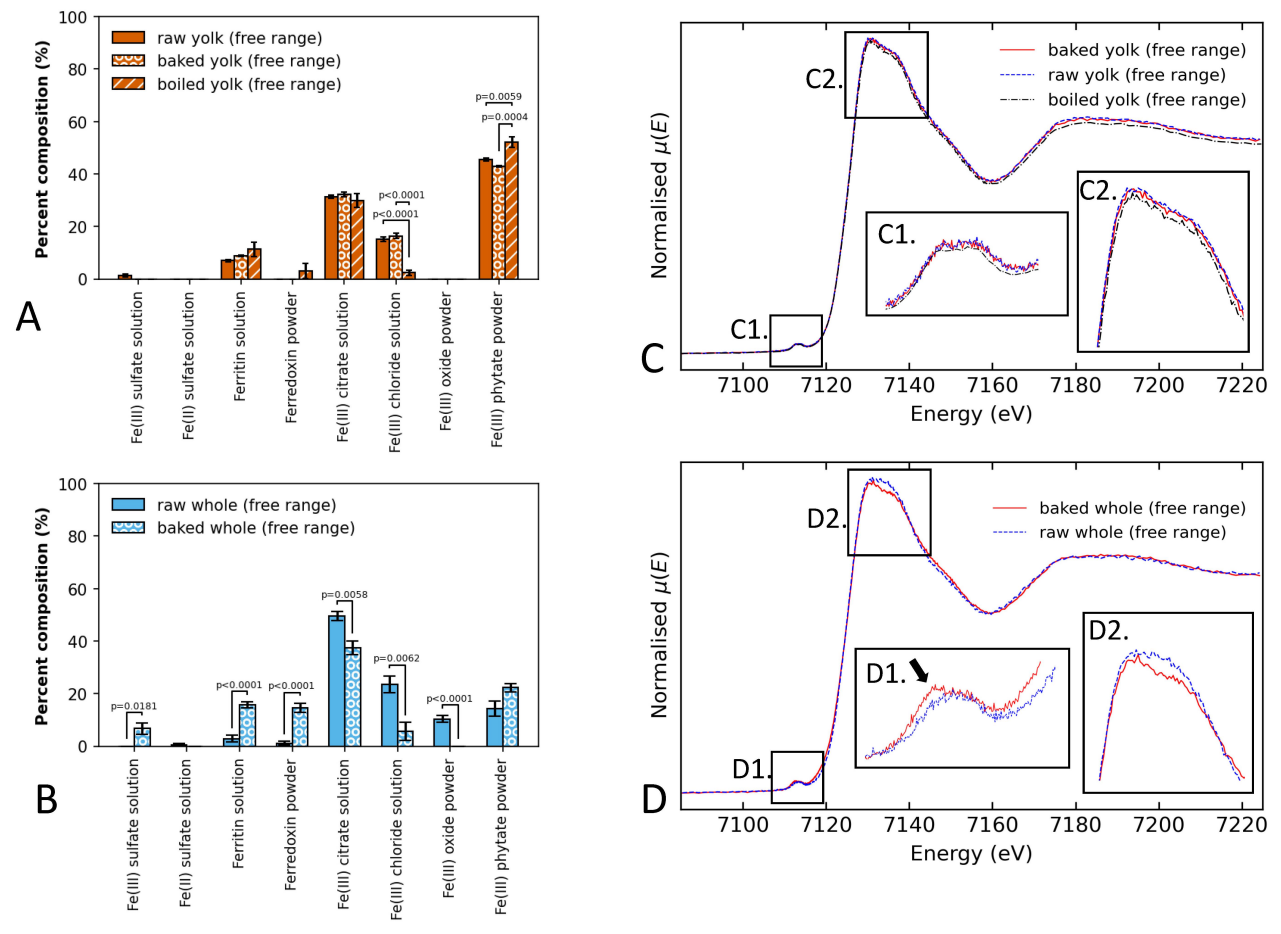
Statistical analysis of LCF determined differences in Fe speciation between preparations of (**A**) egg yolk and (**B**) whole egg. Statistical analysis was undertaken with a One-Way ANOVA and multiple comparison testing when 3 experimental groups were present (i.e., panel (**A**)), or a Student’s *t*-test when only 2 experimental groups were present (i.e., panel (**B**)). One-Way ANOVA *p*-values for Fe(III) chloride and Fe(III) phytate in panel (**A**) were 1 × 10^−7^ and 0.0004, respectively. Error bars are reported as the standard error of the mean. Overlay of examples of Fe K-edge spectra comparing (**C**) free-range freeze-dried yolks from 3 different preparations (raw, boiled, baked) and (**D**) free-range freeze-dried whole eggs from 2 different preparations (raw, baked). Insets (**C1**,**C2**) show a close-up of the (**C1**) pre-edge and (**C2**) white line feature, showing identical pre-edges in both spectra, and very similar white line features. Insets (**D1**,**D2**) show a close-up of the (**D1**) pre-edge and (**D2**) white line feature, showing a shift to lower energy of the pre-edge feature in baked whole eggs (relative to raw whole egg), and a broader and more intense white line feature in raw whole egg (relative to baked whole egg).

Following the observation that mixing egg yolks with egg albumen altered Fe speciation, the effect of different cooking methods on Fe speciation was studied (Figure 4). The results of LCF analysis of Fe speciation in raw, baked, and boiled egg yolks are presented in Figure 4A, with representative spectral fits for raw, baked, and boiled yolks shown in Supporting Information Figure S1A–C. An overlay of the spectra from raw, baked and boiled egg yolks is shown in Figure 4C. Interestingly, the process of cooking had minimal effect on Fe speciation in egg yolks, with few statistically significant differences observed between cooked and raw (Figure 4A). The only difference of note was significantly less Fe resembling the Fe(III) chloride reference spectrum was observed in boiled egg yolks (~2%) compared to raw (15%) or baked yolks (16%), and slightly more Fe resembling Fe(III) coordination to phosphates was observed in boiled eggs (52%) compared to baked (43%) and raw yolk (45%).

In contrast to egg yolks, large differences in Fe speciation were observed between different cooking methods of whole eggs (Figure 4B,D). The process of baking whole eggs resulted in a significant increase in fitting contribution from Fe(III) sulfate (0% to 7%), Fe bound to ferritin (3% to 16%), ferredoxin (1% to 15%), and a decrease in fit contributions from Fe(III) citrate (50% to 37%), Fe(III) chloride (24% to 6%), and Fe oxide (10% to 0%). Interestingly, ferredoxin, which models Fe sulfur compounds, was only detected in baked whole egg samples and was not detected in raw whole egg or any of the yolk samples. Representative spectral fits for raw and baked whole eggs are shown in Supporting Information Figure S1D,E. Interestingly, the changes in Fe speciation observed due to cooking methods, or from simply mixing egg albumen and egg yolk, do not appear to be limited to Fe alone and were also observed for Zn, as presented in Supporting Information Figure S2.

### 3.5. Comparison of Fe Speciation Between Different Laying Environments (Free-Range, Barn, Caged, Organic)

**Figure 5:**
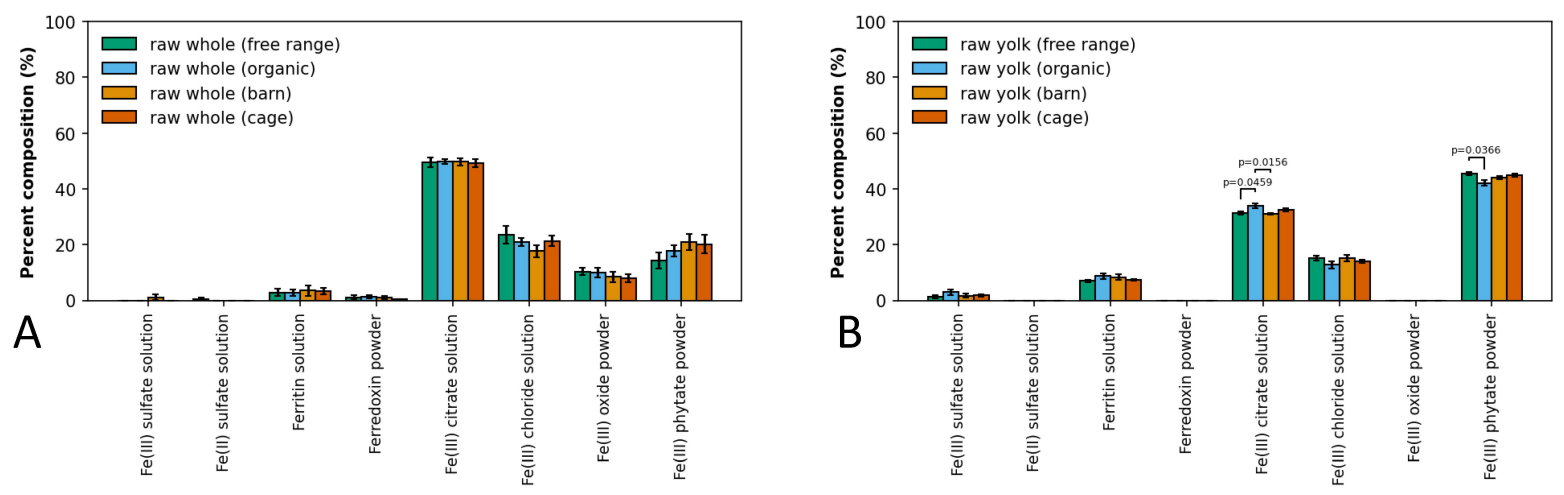
Comparison of Fe speciation in (**A**) whole eggs and (**B**) egg yolks from different housing conditions. Statistical analysis was undertaken with a One-Way ANOVA and multiple comparison testing. One-Way ANOVA *p*-values for Fe(III) citrate and Fe(III) phytate in panel (**B**) were 0.0153 and 0.0401, respectively. Error bars are reported as the standard error of the mean.

Following the results in Section 3.1, which indicated a potential effect of laying environment (free-range, barn, caged, organic) on Fe content in egg yolk (but not whole egg), the effect of laying environment on Fe speciation was investigated. Similar to the total Fe measurement results, there was no significant difference in Fe speciation observed in whole eggs between any of the laying hen housing systems (Figure 5A, Supporting Information Figure S3). Total Fe measurement of egg yolks revealed subtle differences in Fe speciation between yolks from different housing systems (Figure 5B, Supporting Information Figure S3). Specifically, egg yolk from hens reared in free-range housing conditions, which contained relatively more Fe than the organic counterpart samples, contained a slightly higher amount of Fe coordinated to phosphates [modelled by Fe(III) pyrophosphate]. In contrast, egg yolks from the organic housing condition, which contained slightly lower total Fe content than the free-range counterparts, contained slightly more Fe coordinated to carboxylate groups [modelled by Fe(III) citrate complexes]. It should be noted, however, that although statistically significant, the observed differences are small, 45% and 42% for phosphate coordination between free-range and organic egg yolks and 31%, 34%, 31% and 32% for carboxylate coordination (modelled by citrate) in free-range, organic, barn and caged eggs, respectively.

## 4. Discussion

The levels of total Fe in the egg yolk measured in this study (131, 130, 121, 112–85 µg/g freeze-dried egg) equate to 0.82, 0.81, 0.76 and 0.7 mg of total Fe per egg, for free range, barn, caged and organic eggs, respectively. These measurements are based on the assumption that the majority of egg Fe is found in the egg yolk, and the average freeze-dried (i.e., dry) yolk mass measured in this study was 6.2 g per egg. These values agree well with literature values reported for average Fe content per g of dried egg yolk [30]. There was no effect of housing system on Fe content of whole eggs, but housing system had a minor effect on total Fe content in egg yolks, with free-range eggs showing a small, significant increase compared to organic eggs. It should also be noted that the free-range, barn and caged eggs were sourced from the same supplier and laid over the same time period from hens of the same breed and age, which likely reduced variability. In contrast, the organic eggs had to be sourced from a different supplier, meaning hen age and laying period likely differed, potentially contributing to the differences in Fe content observed. It is therefore concluded from this study that the housing system does not result in any nutritionally meaningful differences in egg Fe content.

The failure to detect haem Fe in whole eggs and egg yolks is another important finding from this study. An absence of haem Fe was not unexpected, as it is widely regarded in the literature that chicken eggs are not a nutritionally significant source of haem Fe. However, since chicken eggs are a cell, they do contain mitochondria in the germinal disc, which contain haem proteins. In addition, egg yolks exhibit catalase activity [16,17,18], a haem-containing enzyme, and egg development is supported by blood vessels located on the outside of the egg. The absence of detectable haem Fe in this study indicated that if haem Fe is present in chicken eggs, it occurs at levels too low to have any nutritional significance. When interpreting the above finding, it is important to consider the associated detection limits of XANES spectroscopy, which are reported to be on the order of ppm [59]. The results of this study, therefore, indicate that haem proteins are not present in egg samples at or above ppm levels.

The chemical forms of Fe that were observed within eggs in this study generally agree with the established literature. In particular, the most abundant form of Fe in egg yolks was found to resemble the reference spectra of Fe(III) coordinated to phosphate groups (modelled by Fe(III) phytate), which is consistent with phosvitin as the major Fe-binding protein in egg yolk. The average amount of Fe resembling Fe(III) coordinated to phosphate groups as measured in this study was 45%, which is lower than might be expected, given that the literature indicates 91–95% of egg yolk Fe is bound to phosvitin [25,60]. This difference can possibly be explained by the fact that phosvitin is unlikely to contain a single, homogenous Fe(III) coordination environment. In phosvitin, Fe(III) is reported to be coordinated by two phosphate groups (4 coordinate bonds), which may exist as a tetrahedral complex, or as an octahedral complex with the addition of 5th and 6th coordination bonds arising from coordination to other ligands (e.g., water, organic acids, protein side chains) [49,50]. The variation in coordination of the 5th and 6th ligands to Fe(III) in phosvitin likely results in XANES spectral variance, which is then modelled in part through LCF by spectra other than phytate (e.g., Fe(III) citrates, Fe(III) chlorides, etc.). Therefore, although the average coordination of Fe resembling Fe(III) to phosphates was observed to be 45% in this study, this likely does not translate to there being only 45% of Fe bound to phosvitin. Nonetheless, the results of LCF do agree with the literature, indicating that the major coordination environment of Fe in eggs is Fe(III) coordinated to phosphates.

A key finding of this study was the observation that the speciation of Fe in egg yolks changes upon mixing egg yolk with egg albumen, which indicates that a portion of Fe ligation in egg yolk is labile. Specifically, Fe XANES spectra revealed that the mixing of egg yolks with egg albumen resulted in an increased abundance of Fe speciation reflecting coordination with carboxylate groups and a corresponding decrease in abundance of Fe coordinated by phosphates that resemble the Fe(III) phytate model complex. One mechanism that could account for the observed change in speciation would be the release of Fe(III) from phosvitin following the mixing of egg yolks and egg albumen. Given the well-established thermal and chemical stability of phosvitin, however [25], a decrease in phosphate coordination from 45% to 14% due to the release of Fe(III) from phosvitin seems unlikely. A more plausible explanation is that there was a relative increase in the number of non-phosphate ligands coordinating Fe(III), namely the addition of, or a change of, the 5th and 6th coordination bonds to Fe(III) within phosvitin, which occurs following mixing of egg yolk with egg albumen. Based on the change in Fe speciation observed in this study following mixing of egg albumen and yolk, it is noteworthy that earlier literature in this field, although not explicitly discussed in those papers, also presents data indicating an increase in labile Fe following mixing of egg yolk with albumen [25]. The percentage increase in labile Fe that was observed in the previous studies is relatively low (4–10% of total Fe), although this is possibly still of relevance when considering that often only 30% of the total egg yolk Fe is bioavailable [25,60]. Taking the previous literature (increased labile Fe following mixing egg yolks and egg albumen) and the findings of this study (altered Fe(III) speciation following mixing of egg yolks and egg albumen) together, it would be interesting for future studies to further investigate if the change in Fe speciation that occurs following mixing egg yolks and egg albumen facilitates an increase in Fe bioavailability [25,60].

Another key finding of this study was the observation that cooking method changes Fe speciation. Although the levels of Fe resembling Fe(III) coordinated to phosphates in egg yolks were found to be different between the three preparations (raw, baked and boiled), the magnitude of the changes was small. This is consistent with the literature, which indicates that Fe coordination by phosphates within phosvitin is resistant to heat degradation. There was a notable decrease in Fe resembling Fe(III) coordinated to chloride following boiling of egg yolks, but not in baked egg yolks (relative to raw egg yolks); the reasons for this change are not known. Numerous small but statistically significant changes in Fe speciation were observed when comparing raw and baked whole eggs; however, these changes may simply reflect changes in labile 5th and 6th ligands of phosvitin-bound Fe(III). One interesting feature of Fe speciation observed in baked whole eggs was the detection of Fe resembling Fe coordinated to sulfur, which was modelled by ferredoxin in this study (although noting the actual chemical form of the Fe is almost certainly not ferredoxin). A possible explanation is that, according to the literature, heat induces decomposition of thiol-rich proteins, such as those in egg albumen containing cysteine residues, which in turn promotes the formation of Fe-S complexes in eggs. It would be interesting for future work to now investigate if the increase in Fe-S speciation in cooked whole eggs changes Fe bioavailability.

The effect of housing conditions on Fe speciation in egg yolks and whole eggs closely mirrored the trends seen for total Fe content. No differences in Fe speciation were detected in whole eggs between free-range, barn, caged or organic systems. In egg yolks, several subtle differences were observed that followed the same trend as total Fe content. Organic egg yolks displayed the lowest total Fe content relative to free-range eggs, containing a lesser amount of Fe in a chemical form resembling coordinated to phosphates, with an associated increase in Fe resembling Fe(III) coordinated to carboxylate groups. Given the small magnitude of these differences and the fact that no differences were observed in whole eggs, this study concludes that housing condition does not have a nutritionally significant effect on Fe speciation in chicken eggs.

## 5. Conclusions

In summary, this study concludes that the housing system (free-range, barn, caged, organic) does not affect the total amount or chemical form of Fe found in chicken eggs, and further supports the literature that chicken eggs are not a nutritionally relevant source of haem Fe. A limitation of this study, though, is that although free-range, barn and caged eggs were from the same supplier, hen breed, and age, the organic eggs were not, which should be taken into account when interpreting the results between the different housing systems. Further, it should be acknowledged that normality and homogeneity of variance within the experiment were not assessed in this study. Interestingly, mixing egg yolks with egg albumen and also the choice of cooking method lead to changes in Fe speciation. Future work should expand the XANES spectral library of phosvitin Fe(III) complexes to better characterise the changes to Fe speciation that can occur upon mixing egg yolk with albumen. Although Fe bioavailability was not directly assessed in this study, based on the changes in Fe speciation that was observed after mixing egg yolk with egg albumen followed by cooking, it could be hypothesised that egg preparations that combine yolk and albumen such as scrambled or omelettes may present more bioavailable Fe than methods where egg yolk and albumen are not combined, which should now be specifically assessed in targeted bioavailability studies.

## Data Availability

The original contributions presented in this study are included in the article/Supplementary Materials. Further inquiries can be directed to the corresponding authors.

## Supplementary Materials

The following supporting information can be downloaded at: https://www.mdpi.com/article/10.3390/foods15142452/s1.

## Abbreviations

The following abbreviations are used in this manuscript:

XANES	X-ray Absorption Near-Edge Structure
LCF	Linear Combination Fitting
MP-AES	Microwave plasma atomic emission spectrometry
MEX	Medium-energy X-ray absorption spectroscopy

## References

[1] Shinde P., Ingale S., Choi J., Kim J., Pak S., Chae B. (2011). Efficiency of inorganic and organic iron sources under iron depleted conditions in broilers. Br. Poult. Sci..

[2] Taschetto D., Vieira S.L., Angel C.R., Stefanello C., Kindlein L., Ebbing M.A., Simões C.T. (2017). Iron requirements of broiler breeder hens. Poult. Sci..

[3] Malik Z.I., Ghafoor M.U., Shah S.H.B.U., Abid J., Farooq U., Ahmad A.M.R. (2025). Unlocking iron: Nutritional origins, metabolic pathways, and systemic significance. Front. Nutr..

[4] Warner M.J., Kamran M.T. (2023). Iron deficiency anemia. StatPearls [Internet].

[5] Zhang G.D., Johnstone D., Leahy M.F., Olynyk J.K. (2024). Updating the diagnosis and management of iron deficiency in the era of routine ferritin testing of blood donors by Australian Red Cross Lifeblood. Med. J. Aust..

[6] Kolarš B., Mijatović Jovin V., Živanović N., Minaković I., Gvozdenović N., Kokeza I.D., Lesjak M. (2025). Iron deficiency and iron deficiency anemia: A comprehensive overview of established and emerging concepts. Pharmaceuticals.

[7] Donker A.E., van der Staaij H., Swinkels D.W. (2021). The critical roles of iron during the journey from fetus to adolescent: Developmental aspects of iron homeostasis. Blood Rev..

[8] Lee S., Son Y., Hwang J., Kim M.S., Shin J.I., Yon D.K., Kassebaum N.J. (2025). Global, regional and national burden of dietary iron deficiency from 1990 to 2021: A Global Burden of Disease study. Nat. Med..

[9] GBD 2021 Diseases and Injuries Collaborators (2024). Global incidence, prevalence, years lived with disability (YLDs), disability-adjusted life-years (DALYs), and healthy life expectancy (HALE) for 371 diseases and injuries in 204 countries and territories and 811 subnational locations, 1990–2021: A systematic analysis for the Global Burden of Disease Study 2021. Lancet.

[10] Conrad M.E., Umbreit J.N. (2002). Pathways of iron absorption. Blood Cells Mol. Dis..

[11] Theil E.C. (2004). Iron, ferritin, and nutrition. Annu. Rev. Nutr..

[12] Tako E., Glahn R.P. (2011). Iron status of the late term broiler (Gallus gallus) embryo and hatchling. Int. J. Poul. Sci..

[13] Ems T., St. Lucia K., Huecker M.R. (2017). Biochemistry, iron absorption. StatPearls [Internet].

[14] Piskin E., Cianciosi D., Gulec S., Tomas M., Capanoglu E. (2022). Iron absorption: Factors, limitations, and improvement methods. ACS Omega.

[15] Hurrell R., Egli I. (2010). Iron bioavailability and dietary reference values. Am. J. Clin. Nutr..

[16] Winternitz M., Rogers W. (1910). The catalytic activity of the developing hen’s egg. J. Exp. Med..

[17] Ball H., Cotterill O. (1971). Egg white catalase: 2. Active component. Poult. Sci..

[18] Ball H., Cotterill O. (1971). Egg white catalase: 1. Catalatic reaction. Poult. Sci..

[19] US Department of Agriculture, Agricultural Research Service, Nutrient Data Laboratory (2015). USDA National Nutrient Database for Standard Reference, Legacy.

[20] Caffa I., Proietti E., Turrini F., Borgarelli C., Ferrando M.R., Formisano E., Neri L.d.C.L., Martini D., Angelino D., Tagliabue A. (2025). Nutritional Aspects of Eggs for a Healthy and Sustainable Consumption: A Narrative Review. Food Sci. Nutr..

[21] Bashir L., Ossai P., Shittu O., Abubakar A.N., Caleb T. (2015). Comparison of the nutritional value of egg yolk and egg albumin from domestic chicken, guinea fowl and hybrid chicken. Am. J. Exp. Agric..

[22] Azmandian J., Shamspour N., Azmandian A., Ahmadipour H., Langari T.A. (2025). The Effect of Egg White Meal on Anemia in Patients on Hemodialysis Taking Erythropoietin and Iron Infusion. J. Ren. Nutr..

[23] Kobayashi Y., Wakasugi E., Yasui R., Kuwahata M., Kido Y. (2015). Egg yolk protein delays recovery while ovalbumin is useful in recovery from iron deficiency anemia. Nutrients.

[24] Hallberg L., Rossander-Hulten L., Brune M., Gleerup A. (1992). Calcium and iron absorption: Mechanism of action and nutritional importance. Eur. J. Clin. Nutr..

[25] Albright K., Gordon D., Cotterill O. (1984). Release of iron from phosvitin by heat and food additives. J. Food Sci..

[26] Samaraweera H., Zhang W.G., Lee E.J., Ahn D.U. (2011). Egg yolk phosvitin and functional phosphopeptides. J. Food Sci..

[27] Yilmaz B., Ağagündüz D. (2020). Bioactivities of hen’s egg yolk phosvitin and its functional phosphopeptides in food industry and health. J. Food Sci..

[28] Fijałkowski P., Pryshchepa O., van Eldik R., Pomastowski P. (2025). Ovotransferrin–Multifunctional protein: Structure, bioactivity, and industrial potential. Int. J. Biol. Macromol..

[29] Xing Y., Wang X., Erdenechimeg S., Liao Q., Zang J. (2025). Advances in structure and function of non-heme proteins in food stuffs: A promising dietary iron source for human health. Food Biosci..

[30] Miller J., Nnanna I. (1983). Bioavailability of iron in cooked egg yolk for maintenance of hemoglobin levels in growing rats. J. Nutr..

[31] Réhault-Godbert S., Guyot N., Nys Y. (2019). The golden egg: Nutritional value, bioactivities, and emerging benefits for human health. Nutrients.

[32] Tang D., Wang R., He X., Chen X., Huo X., Lü X., Shan Y. (2021). Comparison of the edible quality of liquid egg with different cooking methods and their antioxidant activity after in vitro digestion. Food Res. Int..

[33] Hernández-Olivas E., Muñoz-Pina S., Andrés A., Heredia A. (2021). Impact of cooking preparation on in vitro digestion of eggs simulating some gastrointestinal alterations in elders. J. Agric. Food Chem..

[34] Cobbinah-Sam E., Ekaette I. (2025). Thermal processing–driven functional transformations of egg components: Albumen and yolk. Food Res. Int..

[35] Frances E.C., Enoch N.N., Johnson O.O., Eziamaka A.-E.C., Ann M.O. (2023). Effects of Processing (Raw, Frying and Boiling) on the Nutritional Composition of White Chicken Eggs. Asian J. Immunol..

[36] Putt R., Brouwers H., Groves P.J., Muir W.I. (2025). Floor Eggs in Australian Cage-Free Egg Production. Animals.

[37] Anderson K. (2009). Overview of natural and organic egg production: Looking back to the future. J. Appl. Poult. Res..

[38] Küçükyılmaz K., Bozkurt M., Yamaner C., Çınar M., Çatlı A., Konak R. (2012). Effect of an organic and conventional rearing system on the mineral content of hen eggs. Food Chem..

[39] Heflin L.E., Malheiros R., Anderson K.E., Johnson L.K., Raatz S.K. (2018). Mineral content of eggs differs with hen strain, age, and rearing environment. Poult. Sci..

[40] Sekeroglu A., Sari H., Mendil D., Sarica M. (2007). Effects of housing systems on some mineral contents of hen’s eggs. Asian J. Chem..

[41] Aisen P., Enns C., Wessling-Resnick M. (2001). Chemistry and biology of eukaryotic iron metabolism. Int. J. Biochem. Cell Biol..

[42] Ackerman C.M., Lee S., Chang C.J. (2017). Analytical Methods for Imaging Metals in Biology: From Transition Metal Metabolism to Transition Metal Signaling. Anal. Chem..

[43] Hirayama T., Nagasawa H. (2017). Chemical tools for detecting Fe ions. J. Clin. Biochem. Nutr..

[44] Hopp M.-T., Schmalohr B.F., Kühl T., Detzel M.S., Wissbrock A., Imhof D. (2020). Heme determination and quantification methods and their suitability for practical applications and everyday use. Anal. Chem..

[45] Tran D., Georgiadis M., DiGiacomo P., Nirschl J., Cobos I., Rosenberg J., Edwards N., Bone S., Webb S., Zeineh M. (2026). Characterizing ferrous versus ferric iron in Alzheimer’s disease using X-ray fluorescence imaging and XANES spectroscopy. J. Alzheimer’s Dis..

[46] Penner-Hahn J.E. (2005). Characterization of “spectroscopically quiet” metals in biology. Coord. Chem. Rev..

[47] Penner-Hahn J.E. (1999). X-ray absorption spectroscopy in coordination chemistry. Coord. Chem. Rev..

[48] Pushie M.J., Pickering I.J., Korbas M., Hackett M.J., George G.N. (2014). Elemental and chemically specific X-ray fluorescence imaging of biological systems. Chem. Rev..

[49] Mangani S., Orioli P.L., Scozzafava A., Messori L., Carloni P. (1994). EXAFS studies of Fe (III)-phosvitin at high metal to protein ratios. BioMetals.

[50] Mangani S., Orioli P., Scozzafava A., Messori L., Stern E.A. (1990). EXAFS investigation on the iron (III) binding sites of hen phosvitin. Inorg. Chem..

[51] De Brier N., Gomand S.V., Donner E., Paterson D., Smolders E., Delcour J.A., Lombi E. (2016). Element distribution and iron speciation in mature wheat grains (*Triticum aestivum* L.) using synchrotron X-ray fluorescence microscopy mapping and X-ray absorption near-edge structure (XANES) imaging. Plant Cell Environ..

[52] Hackett M.J., Ellison G., Hollings A., Colbourne F., de Jonge M.D., Howard D.L. (2021). “A spectroscopic picture paints 1000 words” mapping iron speciation in brain tissue with “full spectrum per pixel” X-ray absorption near-edge structure spectroscopy. Clin. Spectrosc..

[53] James S.A., Roberts B.R., Hare D.J., de Jonge M.D., Birchall I.E., Jenkins N.L., Cherny R.A., Bush A.I., McColl G. (2015). Direct in vivo imaging of ferrous iron dyshomeostasis in ageing Caenorhabditis elegans. Chem. Sci..

[54] Hollings A.L., Lam V., Takechi R., Mamo J.C.L., Reinhardt J., de Jonge M.D., Kappen P., Hackett M.J. (2020). Revealing differences in the chemical form of zinc in brain tissue using K-edge X-ray absorption near-edge structure spectroscopy. Metallomics.

[55] Hollings A.L., Willans M., Lam V., Takechi R., Mamo J.C.L., Mitchell V., de Jonge M.D., Howard D.L., Ellison G., Hackett M.J. (2026). Imaging zinc speciation in the mouse hippocampus with µXANES Spectroscopic mapping. Metallomics.

[56] Asensio G., Hernández-Arriaga A.M., Martín-del-Campo M., Prieto M.A., Rojo L., Vázquez-Lasa B. (2022). A study on Sr/Zn phytate complexes: Structural properties and antimicrobial synergistic effects against Streptococcus mutans. Sci. Rep..

[57] Newville M. (2013). Larch: An analysis package for XAFS and related spectroscopies. J. Phys. Conf. Ser..

[58] Virtanen P., Gommers R., Oliphant T.E., Haberland M., Reddy T., Cournapeau D., Burovski E., Peterson P., Weckesser W., Bright J. (2020). SciPy 1.0: Fundamental algorithms for scientific computing in Python. Nat. Methods.

[59] Matanitobua V.P., Noller B.N., Chiswell B., Ng J.C., Bruce S.L., Huang D., Riley M., Harris H.H. (2007). Using Synchrotron-based X-ray Absorption Spectrometry to Identify the Arsenic Chemical Forms in Mine Waste Materials. AIP Conf. Proc..

[60] Greengard O., Sentenac A., Mendelsohn N. (1964). Phosvitin, the iron carrier of egg yolk. Biochim. Biophys. Acta.

