# Supplementary material for: Characterisation of Iron Content and Speciation in Australian Eggs
Source: Foods. 2026 Jul 10;15(14):2452. doi: 10.3390/foods15142452 (PMC13409603; doi:10.3390/foods15142452)
Supplement: Supplementary file 1 [file foods-15-02452-s001.zip › foods-4353957-supplementary.pdf]

Supporting Information Figure S1

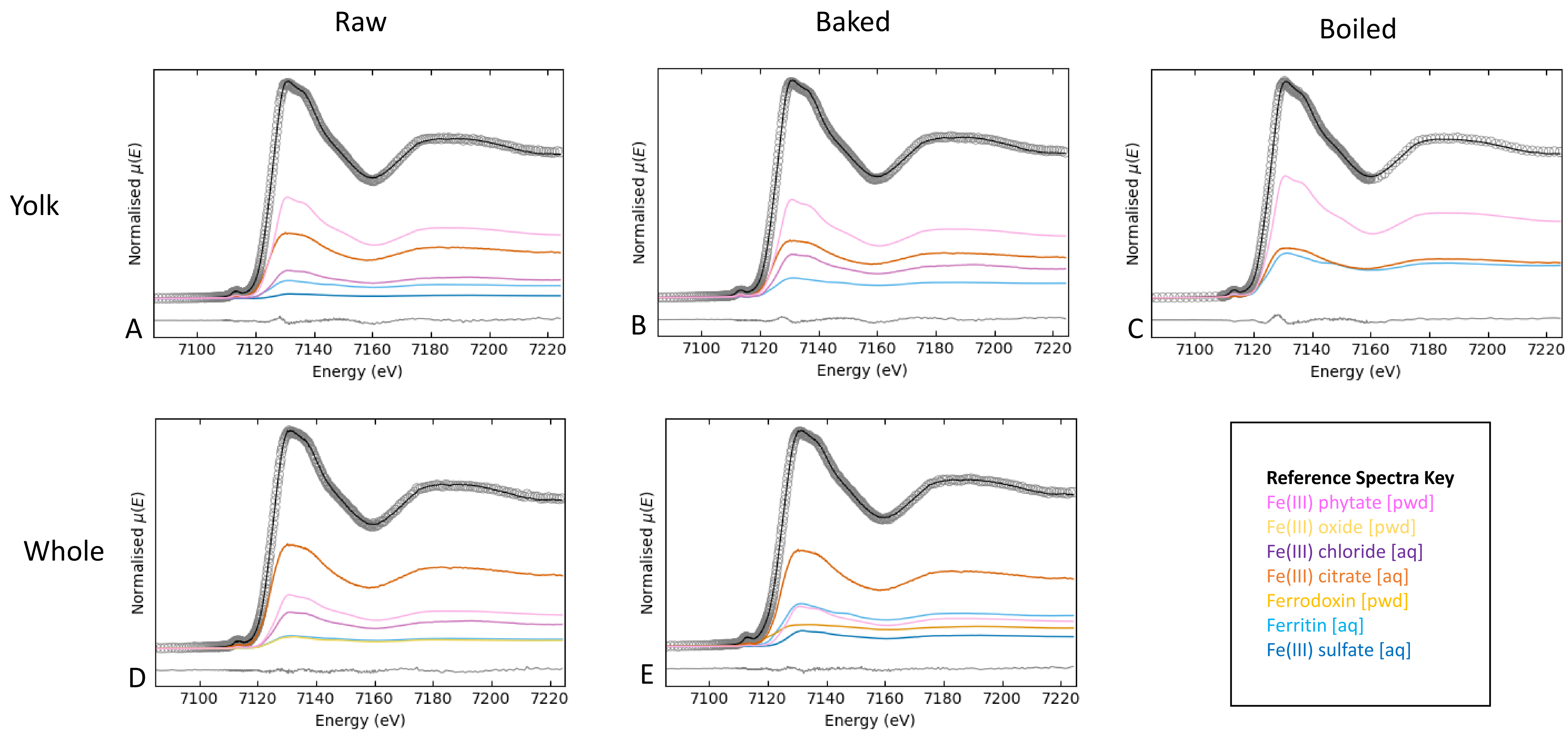

Supporting Information Figure S2

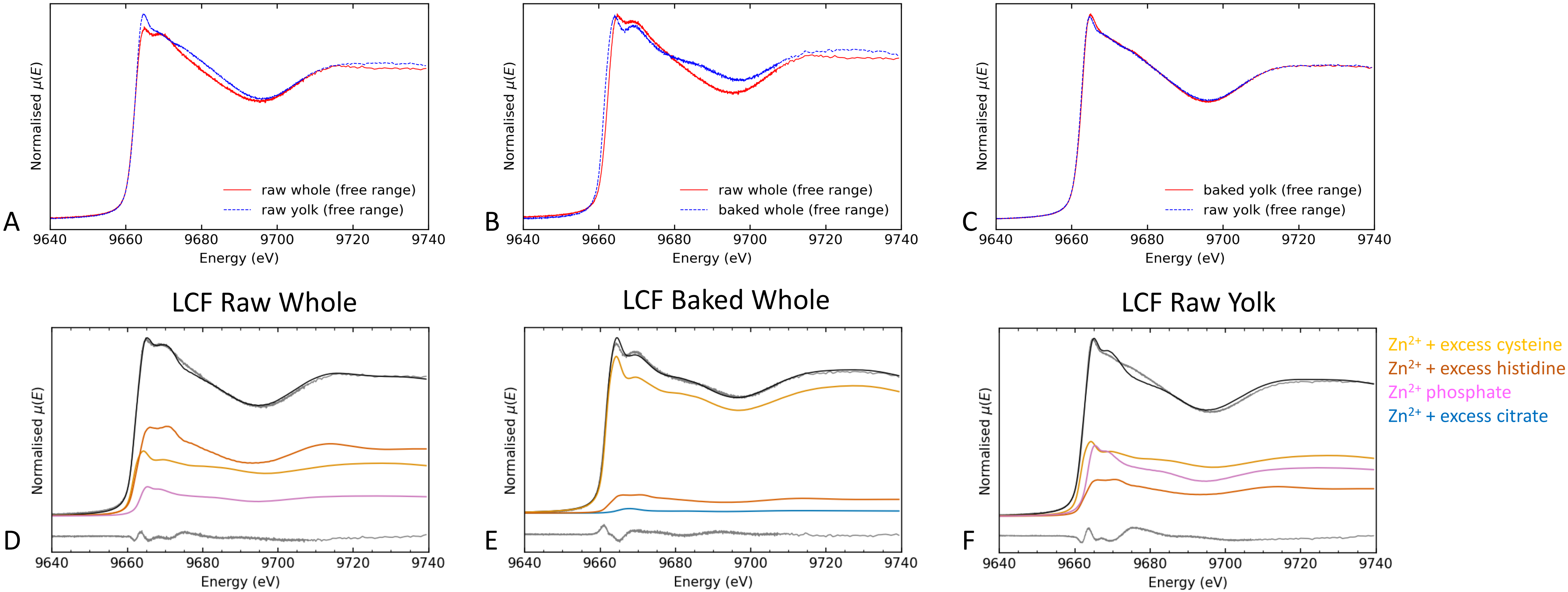

Supporting Information Figure S3

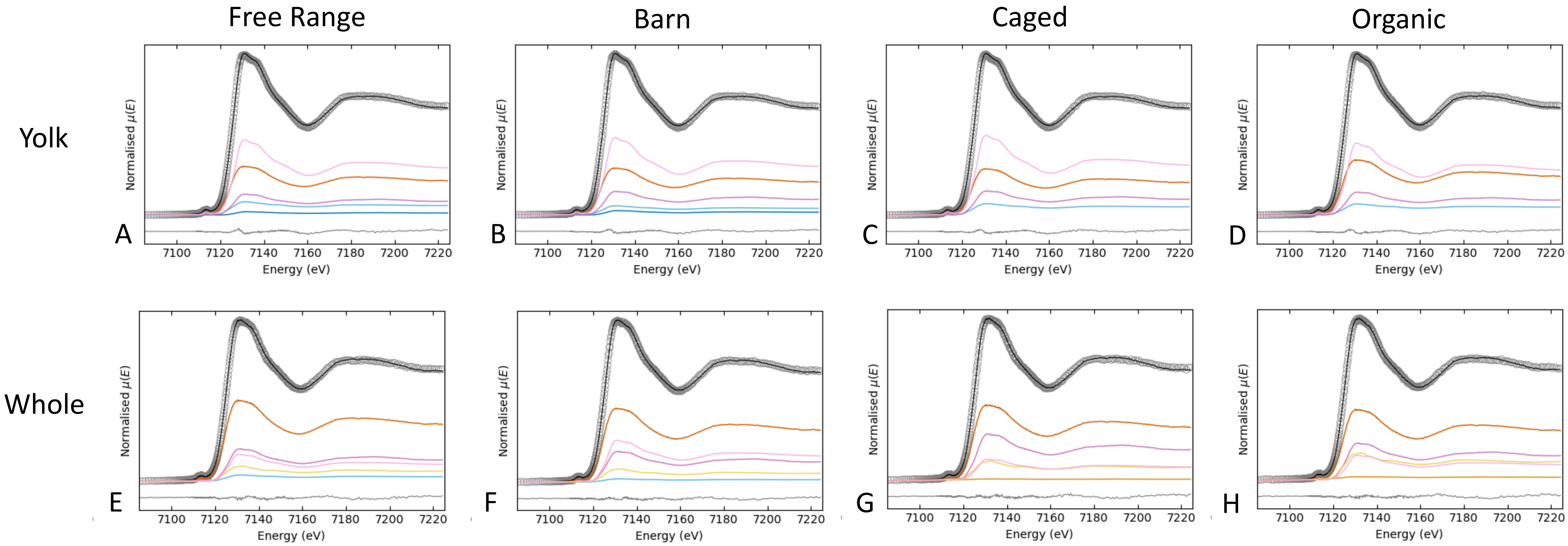

SUPPORTING INFORMATION

Caption: Supporting Information Figure S1

Comparison of representative fits (LCF) of Fe reference spectra to egg yolks (**A-C**) and whole egg (**D-E**) prepared with different cooking methods: Raw (**A, D**), Baked (**B, E**), Boiled (**C**).

Caption: Supporting Information Figure S2

Comparison of Zn K-edge XANES spectra from egg yolks and whole eggs. (**A-C**) Spectral overlays for raw whole egg vs raw yolk (**A**), raw whole egg vs baked whole egg (**B**), baked yolk vs raw yolk (**C**). (**D-F**) Representative fits (LCF) of Zn reference spectra fitted to raw whole egg (**D**), Baked whole egg (**E**), raw yolk (**F**).

Discussion of Figure S2

Although Zn speciation was not a focus of this study, collection of several Zn K-edge XANES spectra from raw egg yolk and whole eggs, and also a comparison between raw and cooked samples, revealed changes in Zn speciation (Supporting Information Figure SI2). Specifically, while Zn coordination was dominated by thiol groups and phosphates in raw yolk, mixing raw yolk with albumen altered speciation such that histidine was the dominant coordinating ligand. Minimal changes in Zn speciation were observed upon baking the yolk, however a large increase in thiol coordination of Zn was seen upon baking the whole egg (mixed yolk and albumen).

Caption: Supporting Information Figure S3

Comparison of representative fits (LCF) of Fe reference spectra to egg yolks (**A-D**) and whole eggs (E-H) from each of the 4 different housing conditions: Free Range (**A, E**), Barn (**B, F**), Caged (**C, G**), Organic (**D, H**).
